# 52739 Trauma Care in Nigeria and Recommendations for Sustainable Improvement to Nigeria’s Trauma Care System: A Systematic Literature Review

**DOI:** 10.1017/cts.2021.574

**Published:** 2021-03-30

**Authors:** Agnes Usoro, Mercy Dickson, Valerie Osula, Angelica K. Ezeigwe

**Affiliations:** 1 Johns Hopkins University School of Medicine; 2 The Ohio State University Wexner Medical Center

## Abstract

ABSTRACT IMPACT: This work highlighs the significant burden of Trauma in Nigeria and will help inform policy decisions on improving Nigeri’s current Trauma care system OBJECTIVES/GOALS: To evaluate trauma care delivery at the pre-hospital, hospital and health systems level in Nigeria in order to identify the burden of trauma, gaps in the delivery of trauma care, and interventions, implemented or recommended, to improve upon the limitations to trauma care delivery. METHODS/STUDY POPULATION: A two-concept search - one being trauma and the other being Nigeria - of the Pubmed (Medline) and Embase databases, in addition to Global Index Medicus and grey literature was performed between September 2018 and September 2019. The search yielded 3,970 articles that underwent title screening and 331 articles that underwent abstract screening. 101 articles were identified for full text screening and the majority were extracted for inclusion into the review. The extracted literature was grouped into 4 categories - articles outlining the burden of trauma in Nigeria, and articles outlining the delivery of trauma care at the pre-hospital, hospital and health systems level. RESULTS/ANTICIPATED RESULTS: Results were classified as an identified challenge or an intervention, recommended or implemented, to address Nigeria’s trauma care system. There was a highlighted need for pre-hospital infrastructure, training of frontline providers, continued competency assessments of frontline providers, in-hospital diagnostic resources, and trauma care surveillance systems to guide health policy. DISCUSSION/SIGNIFICANCE OF FINDINGS: There is a significant burden of trauma in Nigeria. Coordinated interventions and policies at the pre-hospital level, the hospital level, as well as the health systems level are needed in order to address the gaps in Nigeria’s current trauma care system.

